# *Wohlfahrtiimonas chitiniclastica* Infections in two patients with osteomyelitis, China

**DOI:** 10.1016/j.idcr.2025.e02364

**Published:** 2025-09-13

**Authors:** Liqin Meng, Ling Guo, Xin Huang, Jinghan Li, Jinli Bi, Taijie Li

**Affiliations:** a Department of Clinical Laboratory, Wuming Hospital of Guangxi Medical University, Nanning 530199, China; b Department of Clinical Laboratory, The First Affiliated Hospital of Guangxi Medical University, Nanning 530021, China

**Keywords:** *Wohlfahrtiimonas chitiniclastica*, Hydrotalea flava, Osteomyelitis, Chronic wound infection, 16S rRNA gene sequencing, MALDI-TOF-MS

## Abstract

This report presents two cases of human infection caused by *Wohlfahrtiimonas chitiniclastica*, a pathogen associated with chronic osteomyelitis and soft tissue infections. Case 1 involves a 75-year-old male with a long-standing, chronic wound following a right lower leg fracture, which worsened due to inappropriate treatments like "moxibustion" and leech therapy, leading to a severe infection. Despite initial antibiotic therapy with cefoxitin sodium, the infection progressed, resulting in amputation. Case 2 describes a patient with a refractory right plantar ulcer complicated by calcaneal osteomyelitis, who was treated with surgical debridement, followed by antimicrobial therapy based on bacterial culture and susceptibility testing. Both cases were associated with polymicrobial infections, including *W. chitiniclastica*, and required targeted antibiotic therapy. The diagnosis was confirmed using Matrix-Assisted Laser Desorption/Ionization Time-of-Flight Mass Spectrometry (MALDI-TOF MS) and 16S rRNA gene sequencing, highlighting the importance of advanced diagnostic techniques. *W. chitiniclastica* is capable of causing life-threatening infections, including osteomyelitis and myiasis, particularly in patients with poor hygiene or chronic wounds. This study underscores the challenges in identifying emerging pathogens and the necessity for comprehensive diagnostic approaches, including whole-genome sequencing, to improve clinical outcomes and develop effective therapeutic strategies.

## Introduction

Wohlfahrtiimonas chitiniclastica, named for its ability to degrade chitin, is an aerobic, non-motile, non-lactose-fermenting Gram-negative bacterium that produces a light greenish or brownish pigment in culture media[24]. It is primarily isolated from the third instar larvae of Wohlfahrtia magnifica and Chrysomya megacephala, and is considered a potential zoonotic pathogen, frequently associated with parasitic and carrion flies. This bacterium exists as a commensal in these insects, which serve as its primary hosts. While its known hosts include humans, deer, dolphins, and aquatic fish, there are few documented cases of human infection caused by *W. chitiniclastica*([22], [13]).

In this study, antimicrobial susceptibility testing was performed using the Microbial (Enterobacteriaceae) Identification and Susceptibility Analysis System Test Panel (Meihua Bio-Medical). The test principle is based on broth microdilution method, strictly adhering to CLSI standards. The system utilizes pre-configured antibiotic gradient concentrations incubated with bacterial isolates to directly determine the minimum inhibitory concentration (MIC). Testing was performed on the MA120 Microbial Identification and Susceptibility Analysis System (v1.0) (Meihua Bio-Medical).

For bacterial identification, matrix-assisted laser desorption ionization time-of-flight mass spectrometry (MALDI-TOF MS) was employed. Specifically, the Autof MS1000 fully automated microbial mass spectrometry system (Autobio Diagnostics, Zhengzhou, China) was used. This system utilizes a 10-point scoring standard, demonstrating strong discriminatory capability for rare bacterial strains (e.g., W. chitiniclastica). Identification scores ≥ 9.0 were directly reported, while scores below this threshold required additional verification via molecular methods (e.g., 16S rRNA gene sequencing).

The first reported human infection with *W. chitiniclastica* occurred in a patient with an open calf injury[5]. Subsequent case reports from various regions, including North America, Central Europe, and sporadic occurrences in Africa, have highlighted its role as a pathogen in humans[18]. *W. chitiniclastica* infections are most commonly associated with chronic or necrotizing wounds, ulcers, poor hygiene, and underlying cardiovascular conditions[25]. These infections are often polymicrobial and are typically seen in immunocompromised patients, elderly individuals, or those with chronic health conditions[17]. Infections may range from superficial soft tissue infections to severe osteomyelitis and myiasis. The genus Wohlfahrtiimonas was established in 2008 and includes three species: *W. chitiniclastica*, *W. larvae*, and *W. populi*. *W. chitiniclastica* is the most studied species due to its pathogenic potential, while *W. populi* and *W. larvae* are more commonly associated with environmental bacteria and insect-plant symbiosis[24]. *W. chitiniclastica* is transmitted primarily through larvae deposited in wounds and ulcers of vertebrates. It has been reported to cause severe infections, particularly in patients with open wounds or injuries caused by fly bites[4]. The bacterium's pathogenicity is thought to be mediated by its ability to degrade host tissues, often leading to chronic inflammation, tissue necrosis, and secondary infections.

From an epidemiological perspective, while *W. chitiniclastica* infections remain rare, they are becoming more significant due to the increased prevalence of fly vectors and rising incidences of chronic wounds in certain populations[19]. The pathogen is often resistant to tetracyclines, and a multidrug-resistant profile has been reported, complicating treatment options[11]. It is sensitive to several antibiotics, including cefepime, ciprofloxacin, gentamicin, and piperacillin-tazobactam, which should be considered in targeted therapy[23].

This paper reports on a case of *W. chitiniclastica* infection in an open wound, contributing to the understanding of its pathogenicity, the challenges in treatment due to antibiotic resistance, and the importance of early diagnosis and appropriate wound care.

## Case report

### Case1

The first case occurred in 2021, involving a 75-year-old male patient from Wuming District, Nanning City, Guangxi Zhuang Autonomous Region, China, who had long resided in a rural area. The patient had suffered an open comminuted fracture of the right lower leg 52 years earlier due to trauma. He had no recent international travel history nor domestic travel history to other provinces in China. He had received treatment at a local institution (details unavailable), but a small wound, approximately the size of a fingernail, persisted on the anterior aspect of the right tibia, with occasional exudate. Over a year prior to seeking further medical attention, the patient self-administered "moxibustion" therapy, after which the wound became inflamed, with surrounding redness, swelling, and pus accumulation. The patient then sought treatment at a private clinic (details unavailable), where leeches were applied to the wound in an attempt to aid healing. However, this led to the wound progressively ulcerating, expanding, and deepening, ultimately reaching the bone. Necrosis of the surrounding soft tissue followed, exacerbating the condition.

The patient subsequently performed self-administered "wound dressing" at home, which led to further necrosis and the emergence of maggots from the wound (Fig. 2). The patient had a history of recurrent ulceration and persistent wound discharge following open fracture surgery of the right lower leg 50 years ago. Physical examination revealed a circumferential ulcerative wound extending from the mid-calf to the ankle, with significant tissue erosion, necrosis, cauliflower-like granulation tissue, sinus tract formation, and exposed tibia. The surrounding skin exhibited hyperpigmentation and dryness. DR imaging demonstrated bone destruction and lesion formation in the right tibia and fibula, confirming the presence of chronic sclerosing osteomyelitis.

**Fig. 1:**
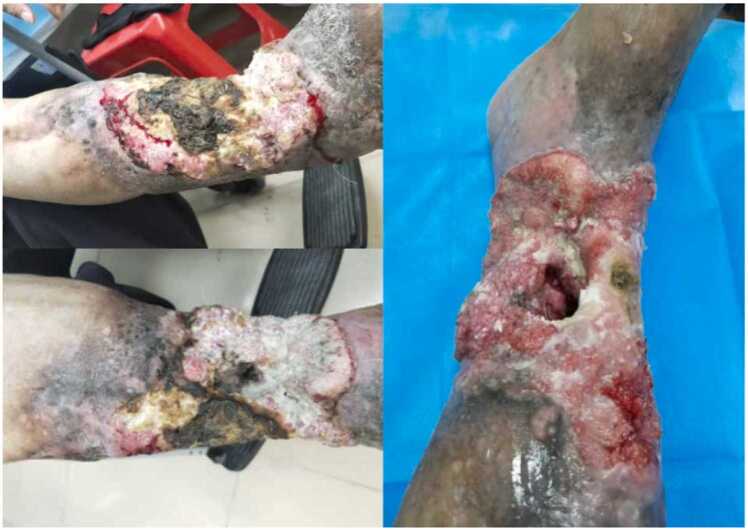
The preoperative photograph of the patient's right lower extremity shows ulcerative erosion of the traumatized tissue. The photo on the left depicts the clinical condition of the infected wound upon admission, while the photo on the right shows the wound after cleaning and larval removal.

**Fig. 2:**
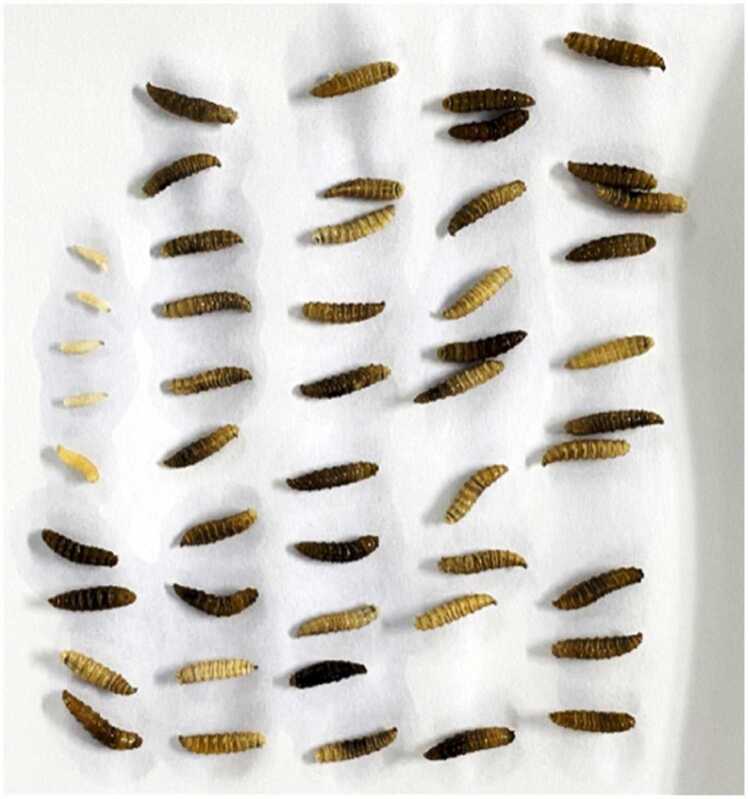
Maggots removed from trauma tissue.

Upon admission, the wound was approximately 30 × 20 cm in size, located on the middle part of the right calf extending to the ankle joint. The wound showed erosion and necrosis of the traumatized tissues, pale red granulation tissue, swelling, cauliflower-like appearance, and yellowish-white purulent exudate adhering to the wound surface. A sinus tract was noted on the medial anterior tibial side of the wound, which extended deep enough to expose the bone. Part of the tibia was visible, including the broken end and necrotic bone. A yellowish-brown medicinal crust was observed on the lateral anterior tibial side of the wound. Swelling was noted on the dorsum of the right foot, with restricted movement in the right ankle joint. The dorsalis pedis artery was weak, blood circulation to the toes was reduced, and the patient reported pain, warmth, and tactile sensation in the affected area (Fig. 1, Fig. 2).

Additional examinations included tibiofibular digital radiography (DR), which revealed chronic sclerosing osteomyelitis of the right tibia and fibula and soft tissue infection of the right calf, along with osteomalacia in the right ankle. Chest Computed Tomography (CT) showed mild thickening and calcification of the left upper pleura, as well as atherosclerosis in the thoracic aorta and coronary arteries. Laboratory results indicated elevated fibrinogen (FIB) at 6.23 g/L (normal range: 2–4 g/L) and C-reactive protein (CRP) at 81.77 mg/L (normal range: <5 mg/L), while leukocyte and neutrophil counts were normal, and liver function tests were within normal limits. The diagnosis included chronic osteomyelitis of the right tibia and fibula and a chronic ulcer of the right lower leg.

To assess for possible squamous cell carcinoma (SCC), a pathological biopsy of the wound was performed. The squamous cell carcinoma antigen (SCC-Ag) level was found to be 14.2 ng/mL, exceeding the normal range and raising suspicion for SCC. Subsequent bacterial cultures of the pus were conducted on MacConkey agar and blood agar plates, which revealed mixed bacterial growth. Identification of the organisms was carried out using Matrix-Assisted Laser Desorption/Ionization Time-of-Flight Mass Spectrometry (MALDI-TOF-MS), followed by targeted antibiotic susceptibility testing. The initial treatment for the patient involved applying silver sulfadiazine ointment to the wound and changing the dressing regularly. Diosmin tablets were prescribed to reduce swelling and prevent thrombosis. Red light therapy was used to enhance microcirculation, while visible blue light was employed to inhibit bacterial growth. The patient's Venous Thromboembolism (VTE) score was 3, indicating an intermediate risk for venous thromboembolism, and oral diosmin tablets were administered as thromboprophylaxis. No antibiotic treatment was initiated initially, as the mixed infection with *Wohlfahrtiimonas chitiniclastica* had not yet been confirmed. After the confirmation of the infection, cefoxitin sodium was prescribed for anti-infective therapy, based on the results of antimicrobial susceptibility testing (see Table 1).

**Table 1:** Results of the documented antibiotic susceptibility tests of W. chitiniclastica.

Antibiotic	MIC(ug/mL) Case1*	Interpretation CLSI	MIC of Case2(ug/mL)	Interpretation CLSI
Amikacin	≤ 4	S	≤ 4	S
Aztreonam	≤ 4	S	≤ 4	S
Ceftazidime	≤ 1	S	≤ 1	S
Chloramphenicol	≤ 8	S	≤ 8	S
Ciprofloxacin	≤ 1	S	≤ 1	S
Sulbactam and Cefoperazone	≤ 4/2	S	≤ 4/2	S
Ceftriaxon	≤ 1	S	≤ 1	S
Cefotaxime	≤ 1	S	≤ 1	S
Doxycycline	≤ 4	S	≤ 4	S
Cefepime	≤ 2	S	≤ 2	S
Gentamicin	≤ 2	S	≤ 2	S
Imipenem	≤ 1	S	≤ 1	S
Levofloxacin	≤ 2	S	≤ 2	S
Minocycline	≤ 4	S	≤ 4	S
Meropenem	≤ 1	S	≤ 1	S
Piperacillin/Tazobactam	≤ 4/4	S	≤ 4/4	S
Tazobactam	≤ 8	S	≤ 8	S
Trimethroprim/Sulfamethoxazole	＞4/76	R	≤ 2/38	S
Ticarcillin	≤ 2/8	S	≤ 2/8	S
Tobramycin	= 4	S	≤ 1	S

* Note: The bacterial strain tested in Case 1 was the DSM18708 reference strain (formerly classified as Wohlfahrtiimonas chitiniclastica prior to 2023, subsequently reclassified as Hydrotalea flava).

After bacterial culture, DNA was extracted and subjected to 16S rRNA gene sequencing. To confirm the identity of the patient isolate, 16S rRNA gene amplification was performed using primer 1492R-sgR, followed by bidirectional sequencing on an ABI 3730xl sequencer (Thermo Fisher Scientific, USA). All sequencing was performed by RuiBio BioTech (Beijing, China) under ISO 13485-certified conditions. The raw sequences were analyzed using BLAST against the NCBI 16S rRNA database (updated January 2023). Sequence analysis using the NCBI 16S rRNA database revealed the following homology: 98.518 % similarity to *Wohlfahrtiimonas chitiniclastica* (NR042554.1) and a 98.090 % similarity to *Wohlfahrtiimonas larvae* (NR133893.1). The sequence corresponding to NR_042554.1 in the results matched the strain *Wohlfahrtiimonas chitiniclastica* DSM 18708 in the NCBI database, which was originally annotated as *W. chitiniclastica*. However, according to the latest DSMZ classification (2023), strain DSM 18708 has since been reclassified as *Hydrotalea flava*[14]. Further identification using Matrix-Assisted Laser Desorption/Ionization Time-of-Flight Mass Spectrometry (MALDI-TOF-MS) confirmed *Wohlfahrtiimonas chitiniclastica* with a high confidence score of 9.558. Additionally, bacterial culture revealed a mixed infection with *Providencia stuartii* and *Wohlfahrtiimonas chitiniclastica*, further supporting the polymicrobial nature of the infection.

After diagnosing a chronic ulcer on the right lower leg, the attending physician performed a biopsy of the ulcerative wound. Using a scalpel, tissue samples were taken from the wound's edge, the cauliflower-like mass within the wound, and the wound center. These samples were then sent for pathological examination. The pathological report revealed papillary proliferation of squamous epithelium and pseudoepitheliomatous hyperplasia in the cauliflower-like mass, along with significant hyperkeratosis and dyskeratosis. However, no definitive evidence of malignancy was found in the wound tissue. Following this, the planned surgical intervention involved debridement of the necrotic tissue and drainage of exudate from the chronic ulcer.

During the debridement of the chronic ulcer on the right lower leg, the resected cauliflower-like ulcerative tissue was sent for pathological analysis. The postoperative pathological report confirmed the presence of well-differentiated squamous cell carcinoma, with a tumor size measuring 7.6 cm × 7.5 cm × 1.2 cm. The pathology confirmed a cutaneous malignant tumor, and the patient was advised to undergo amputation of the right lower limb. Following the amputation surgery and postoperative care, the patient's condition improved, and they were eventually discharged.

### Case2

Another patient was a 72-year-old elderly woman from Nanning City, Guangxi Zhuang Autonomous Region, China, who had long resided in an urban area. The patient had no recent international travel history nor domestic travel history to other provinces in China, and was admitted with recurrent fluid leakage from the right sole for over five years.More than five years before admission, she developed pain and discomfort in the right sole without obvious cause. She subsequently received local injection therapy at a private clinic (details unspecified), after which fluid gradually began to seep from the right foot and progressively spread. Following the onset of symptoms, she underwent surgical treatment at a tertiary hospital in 2017, after which her condition improved, and she was discharged. After discharge, the plantar wound on the patient’s right foot gradually developed increased exudate and continued to expand, although without any accompanying fever or systemic symptoms. Despite management with rest and regular dressing changes, the wound showed no improvement, prompting referral to a tertiary hospital for further evaluation and treatment. Upon physical examination, significant swelling of the right calf’s soft tissue was noted compared to the unaffected side. A 6 cm diameter ulcerative lesion was observed on the plantar surface, extending to the calcaneal level. The wound exhibited abundant purulent discharge and marked edema in the surrounding tissues.

Imaging studies including non-contrast CT and 3D bone reconstruction of the right foot, performed at our hospital, revealed an ulcerative lesion at the plantar heel region and osteomyelitis of the right calcaneus. Laboratory tests on admission showed the following results: procalcitonin (PCT) 0.06 ng/mL, N-terminal pro b-type natriuretic peptide (NT-proBNP) 675.3 pg/mL, hemoglobin (HGB) 89 g/L, neutrophil percentage (NEUT%) 57 %, white blood cell count (WBC) 4.51 × 10⁹/L, urinary white blood cell count (UFWBC) 495.4/μL, microscopic white blood cells (WBC) 45/HP, total protein (TP) 59.1 g/L, albumin (ALB) 30.2 g/L, potassium (K) 3.12 mmol/L, erythrocyte sedimentation rate (ESR) 44 mm/h, activated partial thromboplastin time (APTT) 47.6 s, and fibrinogen (FIB) 4.18 g/L, with other parameters remaining within normal limits.

The patient presented with a refractory right plantar ulcer complicated by calcaneal osteomyelitis and soft tissue infection that had failed to respond to conservative treatment. The surgical management was carried out in two stages: initial radical debridement with ulcer repair and Vacuum sealing drainage (VSD), followed by definitive reconstruction. This included excision of the osteomyelitic focus, medial plantar artery pedicled flap transfer (with intraoperative vascular exploration), and staged flap repair for chronic wound closure. Following the initial surgery, bacterial culture of the purulent discharge identified *Escherichia coli* and *Wohlfahrtiimonas chitiniclastica*, indicating a polymicrobial infection in the right plantar region (antimicrobial susceptibility results are shown in Table 1).

Empirical therapy with ciprofloxacin was initiated prior to susceptibility testing, which was later adjusted to cefazolin sodium postoperatively based on the antibiogram. Although the wound did not achieve complete closure following the initial treatment, repeat cultures of the wound exudate during follow-up identified *Proteus mirabilis*. In accordance with the updated susceptibility profile, cefazolin was discontinued and replaced with piperacillin sodium-tazobactam sodium (4.5 g every 8 h) for targeted antimicrobial therapy.

Ultimately, the patient showed significant clinical improvement, with a reduction in wound size, the absence of systemic symptoms such as fever, and a stable condition. The patient was subsequently discharged.

## Discussion

The two cases of *Wohlfahrtiimonas chitiniclastica* infection presented in this report share several common features. Both patients were elderly (over 70 years old) and had chronic osteomyelitis. Neither of them presented with fever or chills, but both had soft tissue infections in the right calf, complicated by a bacterial mixed infection. Both patients initially sought treatment at private clinics for discomfort in the right foot, but their condition deteriorated before they were referred to a hospital. Laboratory results indicated elevated C-reactive protein (CRP) levels, while leukocyte and neutrophil counts remained within the normal range.

Case 1 was treated with cefoxitin sodium for a postoperative wound infection based on antimicrobial susceptibility testing results. Although partial wound healing was achieved, the pre-existing sinus tract did not resolve completely with antibiotic therapy. Due to progressive tissue necrosis and uncontrolled infection, limb amputation was ultimately required. This highlights the severity of *W. chitiniclastica* infections and their potential complications. Similar cases have been reported globally, including a 2018 case in Japan involving a 70-year-old patient with squamous cell carcinoma infected with *W. chitiniclastica*, who showed improvement after intravenous treatment with cefepime and metronidazole[15]. In 2011, a homeless man in the U.S. developed septic shock and multiple organ failure due to *W. chitiniclastica* infection[1]. Clinical reports confirm the pathogenic potential of *W. chitiniclastica* in humans, with documented infections associated with life-threatening complications([4], [2], [8], [25]).

In China, *W. chitiniclastica* was first isolated in 2012 from *Chrysomya megacephala* at Shanghai Pudong Airport, and its genome sequence was later obtained[3]. Between 2015 and 2022, multiple non-human infections were reported in dairy cattle and zebras in China([9], [21]). However, only one case of human infection has been documented in 2023, involving a diabetic patient with necrotizing fasciitis of the left lower extremity and septic shock[25].

In this study, both patients had *W. chitiniclastica* infections complicated by osteomyelitis. However, the 75-year-old patient from Case 1 had a particularly severe infection, with chronic osteomyelitis leading to squamous cell carcinoma of the surrounding skin and the presence of fly maggots in the wound. Poor hygiene, chronic necrotizing wounds, and osteomyelitis of the foot were key contributors to infection in both cases. Notably, inappropriate prior treatments exacerbated the condition in both patients, leading to the worsening of the infection.

Two similar cases of *W. chitiniclastica* infection have been documented previously. In 2016, South Africa reported the first trauma-associated case involving a 17-year-old male with right arm and shoulder soft tissue infections. The patient was successfully treated with intravenous ceftriaxone (1 g daily) and survived[12]. More recently, in 2022, a 53-year-old male in Belgium presented with a chronic right heel wound complicated by osteomyelitis, polymicrobial infection, and myiasis. This case was managed successfully with amoxicillin/clavulanic acid therapy[6].

Based on domestic and international case reports, effective treatment regimens should be tailored to the patient's condition. For severe cases, particularly those complicated by sepsis or soft tissue infections, initial empirical therapy with β-lactam/β-lactamase inhibitor combinations (e.g., piperacillin-tazobactam) may be considered to control infection progression. Once pathogen identification and antimicrobial susceptibility results are available, therapy should be de-escalated to more targeted antibiotics (such as cefepime, ciprofloxacin, or gentamicin), with adjustments made based on susceptibility profiles to minimize resistance development([12], [8], [10]). Combined wound debridement, chlorhexidine irrigation, and pulsed lavage can effectively eliminate infection sources and necrotic tissue, particularly insect residues like maggots. In advanced cases, surgical intervention, including excision of infected soft tissue or bone, may be required.

In our clinical experience, prompt hospital referral upon detecting *W. chitiniclastica* infection is crucial to prevent disease progression. For accurate species identification, matrix-assisted laser desorption ionization time-of-flight mass spectrometry (MALDI-TOF MS) and 16S rRNA sequencing are considered the gold-standard diagnostic methods[7]. While automated identification platforms like Vitek 2 and API 20 NE are widely used, they often fail to identify emerging pathogens such as *W. chitiniclastica*. These systems have been known to misidentify *W. chitiniclastica* as more common Gram-negative bacteria, such as *Acinetobacter lwoffii*, which can lead to incorrect antibiotic selection and clinical deterioration([7], [16]). Therefore, for polymicrobial infections or unidentified pathogens, we recommend prioritizing 16S rRNA gene sequencing and MALDI-TOF MS for confirmatory diagnostics.

The 2023 reclassification of *W. chitiniclastica* DSM 18708 as *Hydrotalea flava* poses diagnostic challenges for clinical isolates[20]. Our isolate met all the criteria for *W. chitiniclastica* infection (MALDI-TOF MS score >2.3, necrotizing wound phenotype) despite showing 98.518 % 16S rRNA similarity to *H. flava* (NR_042554.1). Definitive resolution of this issue requires whole-genome sequencing for ANI/dDDH analysis, combined with phenotypic confirmation through chitinase testing (positive in *W. chitiniclastica* but negative in *H. flava*) and formal strain deposition. Clinically, the isolate displayed pathognomonic features such as pyogenic necrosis, characteristic tissue invasion patterns, and cefoxitin resistance (MIC >32 μg/mL), affirming its identity as *W. chitiniclastica*, irrespective of recent taxonomic revisions[11]. This case either represents a novel genomic variant requiring further taxonomic clarification or highlights the limitations of 16S-based classification for proteobacterial pathogens, underscoring the need for polyphasic approaches when diagnosing emerging infections.

Furthermore, we compared the performance of MALDI-TOF MS with other bacterial identification methods to assess identification accuracy. The results showed that MALDI-TOF MS and 16S rRNA gene sequencing achieved highly consistent identification results for Wohlfahrtiimonas chitiniclastica (log scores all >2.3), while traditional automated identification systems such as Vitek 2 and API 20 NE frequently produced misidentifications (with a 96 % probability of incorrectly identifying W. chitiniclastica as Acinetobacter lwoffii). Notably, when MALDI-TOF MS was used for verification after bacterial culture isolation, its identification accuracy was significantly superior to conventional biochemical identification methods (with stable log scores of 2.229–2.296 in triplicate testing). Based on the data from this study, we recommend adopting a strategy combining MALDI-TOF MS with 16S rRNA sequencing for suspected rare bacterial infection cases to overcome the limitations of traditional phenotypic identification methods and ensure the reliability of test results.

In summary, the combined application of MALDI-TOF MS and 16S rRNA sequencing ensures reliable identification of rare pathogens such as *W. chitiniclastica*. This complementary approach not only enhances diagnostic accuracy but also helps resolve taxonomic ambiguities, consistent with international best practices.

## Conclusion

This study presents a case of osteomyelitis identified as *Wohlfahrtiimonas chitiniclastica* using MALDI-TOF MS. Despite the 98.518 % 16S rRNA similarity to the reclassified *Hydrotalea flava* DSM 18708 (NR_042554.1), comprehensive clinical and laboratory evidence supports its classification as a pathogenic strain of *W. chitiniclastica*. Future studies should employ whole-genome sequencing to clarify its phylogenetic position. Chronic osteomyelitis infections caused by *W. chitiniclastica* require prompt, comprehensive management. Preventive measures should focus on maintaining wound hygiene, early medical intervention, and avoiding unsupervised treatment, especially for chronic wounds. Future research should further investigate the epidemiological characteristics of *W. chitiniclastica* and its relationship with the host and the environment. Additionally, optimizing rapid and efficient diagnostic techniques, advancing antibiotic susceptibility studies, and developing effective therapeutic strategies to combat drug resistance should be prioritized.

## Author Contributions

LM and LG contributed equally to this work, including case collection, laboratory testing, data analysis, and drafting of the manuscript. XH and JL assisted in laboratory experiments and data interpretation. JB participated in clinical data collection and analysis. TL conceived and designed the study, supervised the research, critically revised the manuscript, and acquired funding. All authors reviewed and approved the final version of the manuscript.

## CRediT authorship contribution statement

**Taijie Li:** Methodology, Investigation, Formal analysis. **Jinghan Li:** Methodology, Investigation. **Jinli Bi:** Methodology, Investigation. **Ling Guo:** Writing – review & editing, Writing – original draft, Data curation, Conceptualization. **Xin Huang:** Validation, Supervision, Data curation. **Liqin Meng:** Writing – review & editing, Writing – original draft, Formal analysis, Data curation, Conceptualization.

## Funding

This research was supported by the 10.13039/100012547Natural Science Foundation of Guangxi Zhuang Autonomous Region (2023GXNSFAA026184)，Guangxi Zhuang Autonomous Region (2025GXNSFAA069134) and Guangxi appropriate technology development and application project (S2022122).

## Declaration of Competing Interest

The authors declare that they have no competing financial or non-financial interests related to this work. All authors must disclose any affiliations with organizations with a financial or non-financial interest in the subject matter discussed in the manuscript.
